# Mechanical Properties and Brittle Behavior of Silica Aerogels

**DOI:** 10.3390/gels1020256

**Published:** 2015-12-10

**Authors:** Thierry Woignier, Juan Primera, Adil Alaoui, Pascal Etienne, Florence Despestis, Sylvie Calas-Etienne

**Affiliations:** 1 Institut Méditerranéen de Biodiversité et d’Ecologie marine et continentale (IMBE), Aix Marseille Université, CNRS, IRD, Avignon Université, UMR CNRS 7263, 13397 Marseille, France; 2 IRD UMR 237-Campus Agro Environnemental Caraïbes-B.P. 214 Petit Morne, 97232 Le Lamentin, Martinique, France; 3 Departamento de Fisica, FEC, LUZ, 4011 Maracaibo, Venezuela; E-Mail: juan.primera2009@gmail.com; 4 Escuela Superior Politécnica del Litoral (ESPOL) Facultad de Ciencias Naturales y Matemáticas, Departamento de Física, Campus Gustavo Galindo Km 30.5 Vía Perimetral, P.O. Box 09-01-5863, 090150 Guayaquil, Ecuador; 5 Faculté des Sciences et Techniques de Tanger, B.P. 416, 90000 Tanger, Marroco; E-Mail: pr_alaoui@yahoo.fr; 6 Laboratoire Charles Coulomb, Université Montpellier 2, Place E. Bataillon, 34095 Montpellier Cedex 5, France; E-Mails: pascal.etienne@umontpellier.fr (P.E.); florence.despetis@umontpellier.fr (F.D.); sylvie.etienne@umontpellier.fr (S.C.-E.)

**Keywords:** aerogels, porous glasses, mechanical properties, elastic properties, toughness, weibull statistic, stress corrosion effect

## Abstract

Sets of silica gels: aerogels, xerogels and sintered aerogels, have been studied in the objective to understand the mechanical behavior of these highly porous solids. The mechanical behaviour of gels is described in terms of elastic and brittle materials, like glasses or ceramics. The magnitude of the elastic and rupture modulus is several orders of magnitude lower compared to dense glass. The mechanical behaviours (elastic and brittle) are related to the same kinds of gel characteristics: pore volume, silanol content and pore size. Elastic modulus depends strongly on the volume fraction of pores and on the condensation reaction between silanols. Concerning the brittleness features: rupture modulus and toughness, it is shown that pores size plays an important role. Pores can be considered as flaws in the terms of fracture mechanics and the flaw size is related to the pore size. Weibull’s theory is used to show the statistical nature of flaw. Moreover, stress corrosion behaviour is studied as a function of environmental conditions (water and alcoholic atmosphere) and temperature.

## 1. Introduction

Many of the studies of mechanical behaviour of porous media are motivated by the question: How are effective macroscopic parameters such as the mechanical properties influenced by the microstructure geometry of the media? The macroscopic physical properties of porous media depend on the microstructural information including the volumetric fraction of each of the phases present [1,2]. Theoretical and computational studies have addressed the topic using percolation elastic networks, scaling concepts, and simulation models of elastic phenomena [3,4,5,6,7].

In the case of materials like porous glass (and ceramics), general agreement is that porous materials exhibit a brittle and elastic behaviour, but their mechanical characteristics are lower than dense materials due to the lower connectivity and to porosity of the network [8]. A new kind of porous glass (dried gels) has been studied in the last decades. These materials are not prepared using the classical melting processes but they are synthesised by sol-gel process [9]. The porous structure of the dried gels results from the aggregation and/or sintering of oxides particles (SiO_2_, ZrO_2_, B_2_O_3_, Al_2_O_3_, TiO_2_ ...).

In the family of gels, silica aerogels have known an increasing interest in different fields from the applications as specific materials to the fundamental research. Silica aerogels have peculiar physical properties such as large specific surface area, very low sound velocity, low thermal and electrical conductivity and fractal structure [10,11,12,13,14]. These features are essentially due to the very large pore volume, which can be easily tailored during the gel synthesis [9,14].

The knowledge of the mechanical properties of gels and aerogels is also clearly of interest for theoretical research. The mechanical properties in relation to the structure can be experimentally studied over the whole range of porosity (0%–99%). In the literature, the elasto-mechanical behavior is tested either by sound velocity measurements [15,16] or static techniques like uniaxial compression [17], diametric compression [18] and three point bending [19,20,21]. Silica aerogels are brittle materials like glass, the stress-strain relation evolves like a common elastic material toward a “catastrophic” fracture under a tension load.

For brittle materials, the strength is strongly dependent on the presence of flaws, which act as stress concentrators [22,23,24]. The most relevant feature of brittle materials is their toughness [23] which characterizes the flaw propagation. The knowledge of the rupture modulus and toughness allows the calculation of the critical flaw size [24] responsible for the fracture. In the literature [19,21], the theory of linear elastic fracture mechanics has been applied to measure the toughness and defect size of silica gels. The pores could be considered as an integral part of flaws responsible for the failure of this brittle material.

In this review, we measure and characterize the mechanical behavior of sets of aerogels. The elastic and mechanical properties of porous materials are strongly dependent on the load bearing fraction of solid and thus on the bulk density. We use different ways to synthesize sets of samples with a tailored pore volume (bulk density), for example by varying the volumetric ratios of the used silane monomers: tetramethoxysilane (TMOS) or by collapsing the porosity by sintering and controlled drying.

## 2. Results and Discussion

### 2.1. Influence of Synthesis and Process Parameters on the Mechanical Properties of Aerogels

It is obvious that the poor mechanical properties of aerogels are due to the large pore volume which characterizes these materials. Consequently, the increase of the bulk density (decrease of the pore volume) will generally improve their mechanical properties. Simple ways to decrease the pore volume are usually done by higher monomer content, by a drying process which collapse porosity or by sintering.

#### 2.1.1. The Silane Concentration

**Figure 1:**
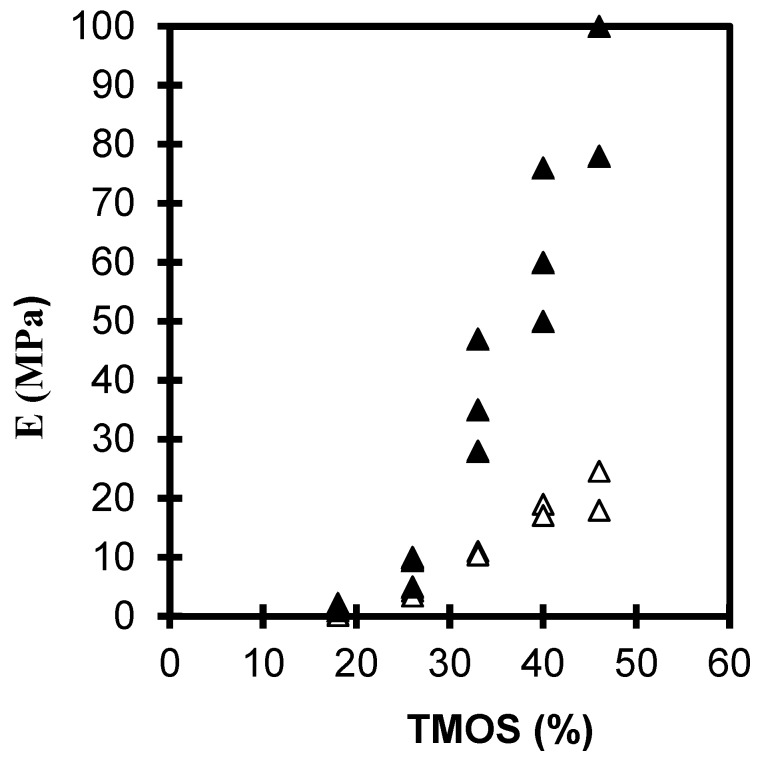
Evolution of E *versus* the TMOS content for neutral (▲) and basic aerogels (Δ).

**Figure 2:**
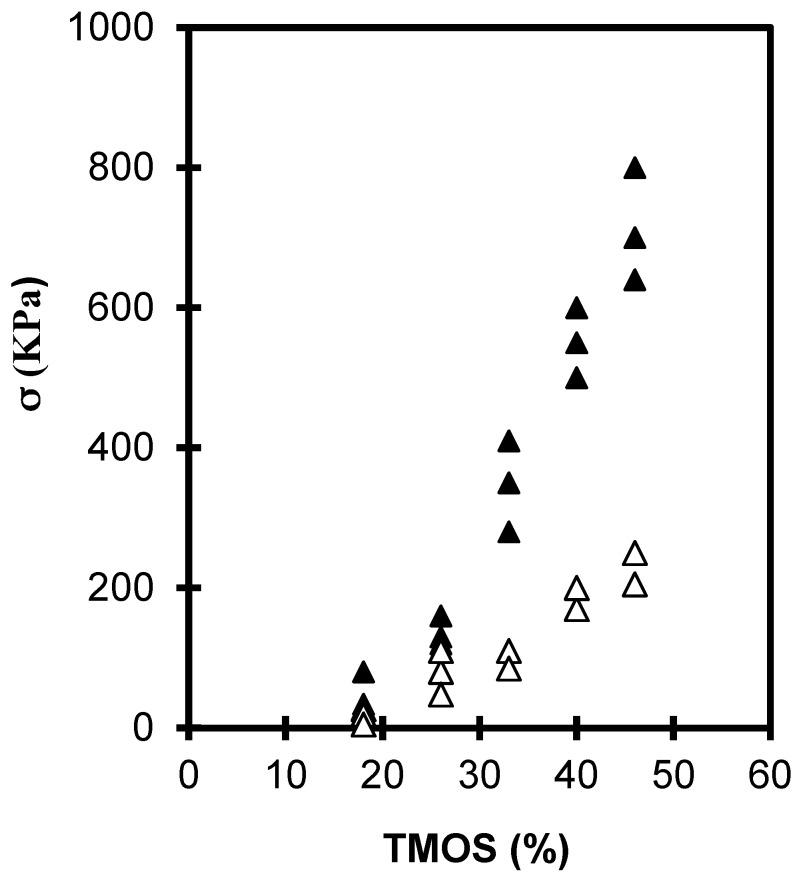
Evolution of σ *versus* the TMOS content for neutral (▲) and basic aerogels (Δ).

Figure 1 and Figure 2 shows the evolution of the elastic young’s modulus (*E*) and the rupture modulus (σ) as a function of the TMOS percent in the case of neutral or basic catalysis (see “Experimental section”). For the 2 sets of samples studied *E* and σ increases by almost one order of magnitude with the TMOS content studied. The figures shows that the mechanical properties of neutral aerogels are higher than those of the basic set because of a structure more reticulated in the case of neutral aerogels [9,14,20]. Neutral gels have a higher bulk density than base catalyzed gels (Table 1).

**Table 1:** Bulk density *versus* tetramethoxysilane (TMOS) content for the basic and neutral sets.

TMOS (%)		Bulk Density, ρ (g·cm^−3^)
Basic set	18	0.1
	26	0.14
	33	0.17
	40	0.21
	46	0.24
Neutral set	18	0.11
	26	0.15
	33	0.23
	40	0.28
	46	0.32

Because the mechanical strength (σ) is strongly dependent on the presence of flaws, the most relevant feature of aerogels is their toughness (*K*_IC_). In order to check if the critical flaw size depends on the porosity and pore size, we measured the toughness for sets of aerogels with different porosities.

**Figure 3:**
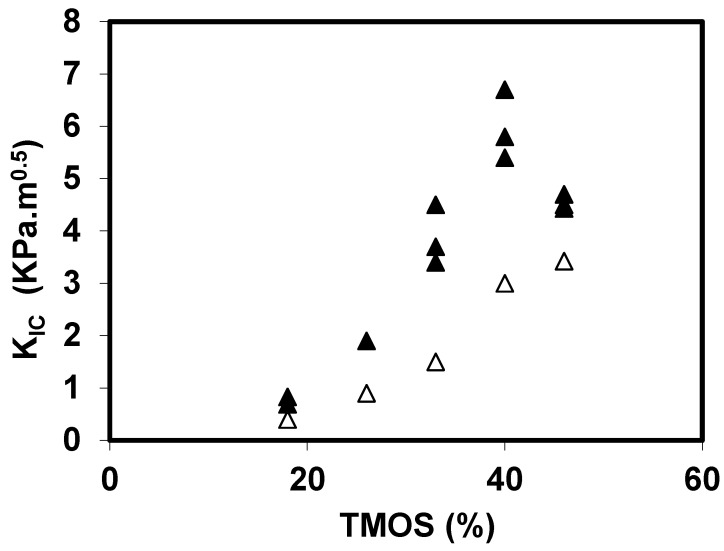
Toughness evolution for the neutral (▲) and basic aerogels (Δ).

Figure 3 shows the evolution of *K*_IC_ for the basic and neutral aerogels, as a function of the TMOS content. The toughening of the two sets of materials is directly related to the increase of the TMOS content. We note that the toughness of the basic set is slightly lower than those of the neutral set and confirms the result previously measured on *E* and σ.

**Figure 4:**
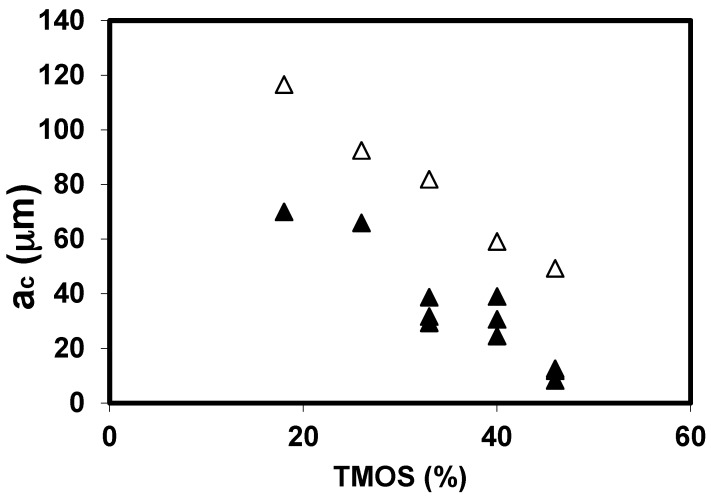
*a*_C_ evolution for the neutral (▲) and basic aerogels (Δ).

From the knowledge of the toughness and of the rupture modulus (*K*_IC_ and σ), we have calculated the critical flaw size, *a*_C_ [16]. The *a*_C_ evolution as a function of the TMOS for the neutral and basic sets is reported in Figure 4, and the general trend is a *a*_C_ decrease with the TMOS content. As suggested before, a correlation could exist between *a*_C_ and the evolution of the pore size. It has been already shown that when the TMOS content increases, the average pore size decreases and the pore size distribution curve shrinks. A previous study [25] has shown that, for the two sets of aerogels, the macroporous volume decreases strongly when the TMOS % increases. If we try to relate the a_C_ change with the textural change, we observe that *a*_C_ follows the size change of the largest pores (macropores).

**Figure 5:**
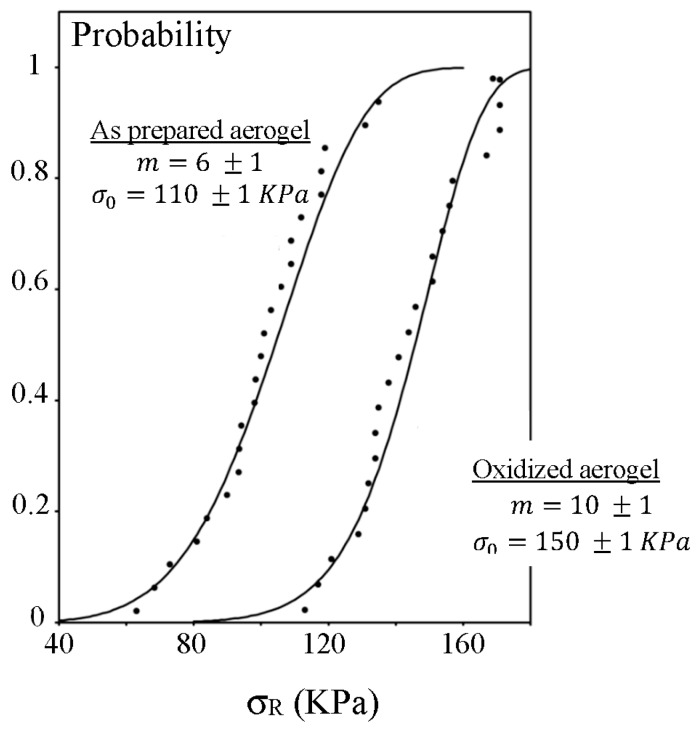
Cumulative failure probability distributions for basic aerogels with 40% TMOS.

For brittle materials, the broad scattering of the mechanical strength values is attributed to the statistical nature of flaws. The sample strength distribution could be analyzed using the Weibull’s statistic (see “Experimental section”). Because of a texture property modification under the oxidizing heat treatment, *a*_c_ flaw distribution is suspected to change. The Figure 5 shows Weibull’s analysis for non-oxidized (called as prepared) and oxidized aerogels.

The value of *m* for oxidized aerogels is higher than that found on as prepared ones, indicating that the flaw distribution appears narrowed, as prepared aerogels contain a large amount of organic species which can be removed by an oxidizing heat treatment. After oxidation, the surface contains a high concentration of OH active groups which undergoes formation of new siloxane bonds by condensation of SiOH groups and probably a collapse of the smallest flaws. The calculation showing that the σ*_o_* value is also increased by a factor 1.4 confirms this hypothesis.

#### 2.1.2. Sintering and Drying Process

**Figure 6:**
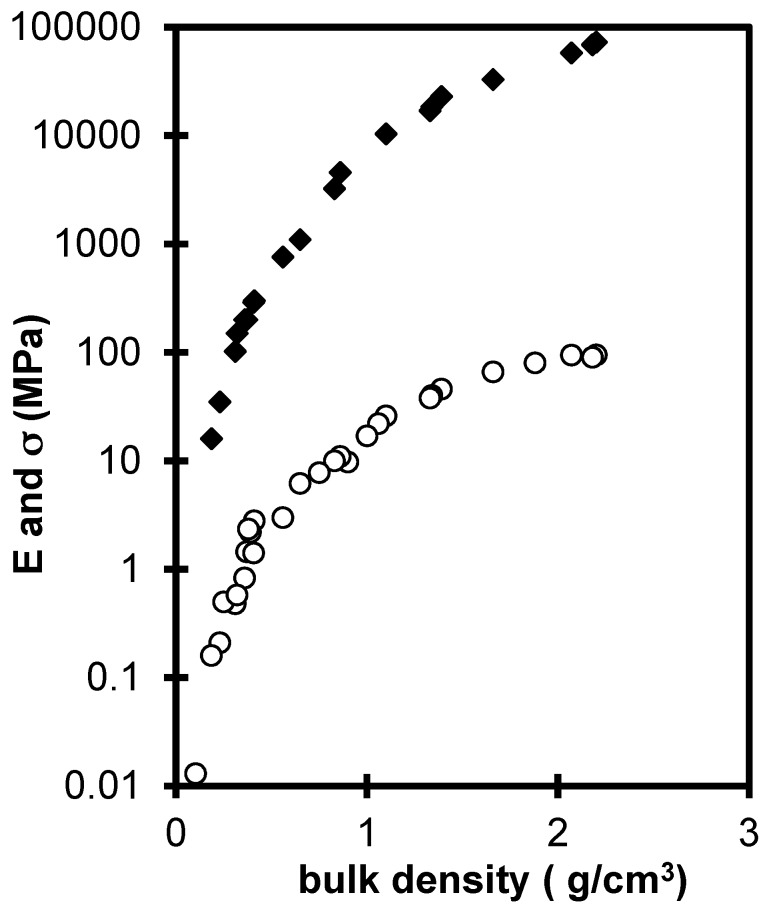
*E* (♦) and σ (ο) evolution for sintered aerogels *versus* the bulk density.

Aerogels can be sintered and transformed in dense silica glasses by a heat treatment close to 1000 °C [26]. During these treatments, the microstructure of the aerogel is changed and the mechanical properties are enhanced. Figure 6 collects the evolutions of the Young (*E*) and the rupture moduli (σ) as a function of the density produced by sintering. The main feature of these curves is the very large increase (10^4^–10^5^) of the elastic and mechanical properties over the bulk density range. The biggest part of the strengthening arises in the density range 0.1–1 g·cm^−3^, the aerogel strengthens and finally the mechanical features of the fully dense material are identical to those of conventional silica glass. In this density range, the strengthening of the material is directly related to the decrease of the pore volume but also, at a given density, to structural changes.

The *E* and σ values of sintered aerogel are higher than for neutral aerogels having the same bulk density [20], we can conclude that the heat treatment has induced an increase of the connectivity or of the size of the necks between particles. Organic species and silanol groups are replaced by new siloxane bonds increasing the connectivity and thus the mechanical features. The sintering reduces the whole sample volume eliminating the macro and microporosity [27,28]. Besides the pore elimination, the heat treatment has structural effects: it increases the network connectivity and shrinks the pore size distribution.

**Figure 7:**
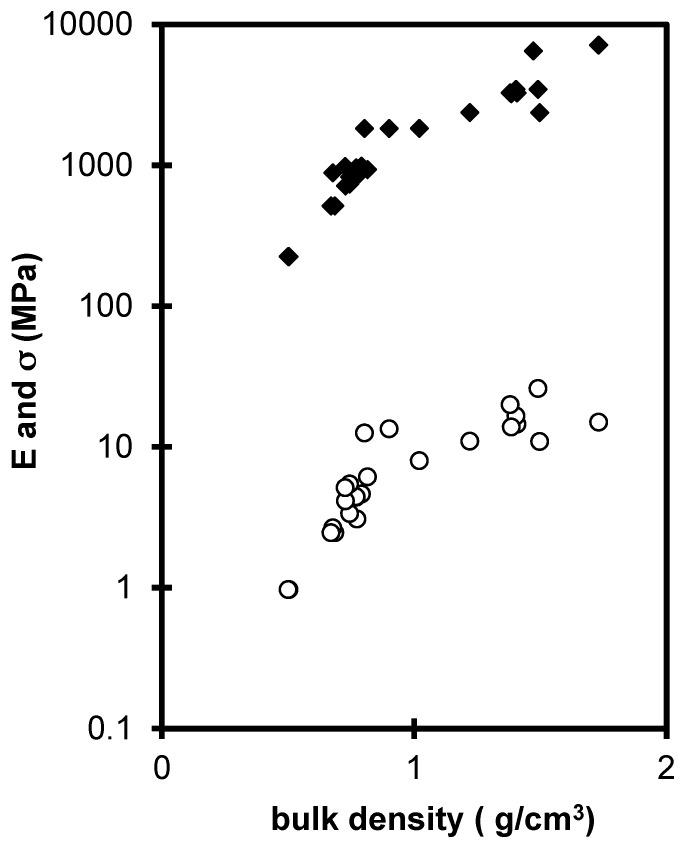
*E* (♦) and σ (ο) evolution for the xerogels *versus* the bulk density.

Finally, the drying process obviously has an effect on the mechanical properties of the gels. When the drying is operated at ambient temperature by evaporation [9], capillary stresses will collapse the gels structure and eliminate porosity. Figure 7 collects the evolutions of the Young modulus (*E*) and the rupture modulus (σ) as a function of the xerogels density produced by the porosity collapse. The stiffening and the strengthening of the material is directly related to the increase of density (decrease of the pore volume).

From the comparisons of the mechanical properties of the xerogels set with aerogels’ mechanical features extrapolated to the same density range, it appears that xerogels have lower mechanical properties than sintered aerogels (Figure 7). The xerogel network is likely damaged by the drying stresses. During drying, the xerogel is submitted to compression force which tends to eliminate the larger pores [9,29] and the pores size distribution shift up to the lower pores [30]. The literature has shown that compression stresses could induce damage in the structure (loss of connectivity) even if the samples are not broken and look to be without cracks [31]. The assumption of the structure damage is deduced from the lower mechanical properties of the xerogels in the density range 1–1.6 g·cm^−3^. It is noteworthy, that *E* and σ increase only slightly in the large density range 1–1.6 g·cm^−3^ corresponding to a large loss of pores (60% of porosity). We should expect a large stiffening and strengthening, but to obtain such high densities by drying, large compression stresses are necessary, which leads to the breakage of links at a microscopic scale and weakens the whole solid structure.

**Figure 8:**
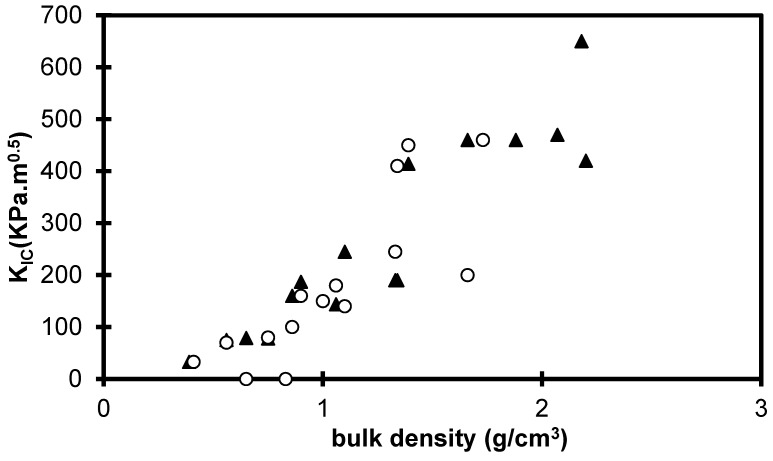
*K*_IC_ evolution for the xerogels (ο) and sintered aerogels (▲) *versus* the bulk density.

Figure 8 shows the evolution of *K*_IC_ for sintered and xerogels materials as a function of the bulk density. The reported values increase by a factor 10^3^ towards those measured on dense silica glass (0.7–1.5 MPa × m^0.5^) [23]. Concerning the comparison between the sintered and the xerogels sets, the difference seems less important than the one measured on *E* and σ. This result will be discussed in terms of critical flaw size.

**Figure 9:**
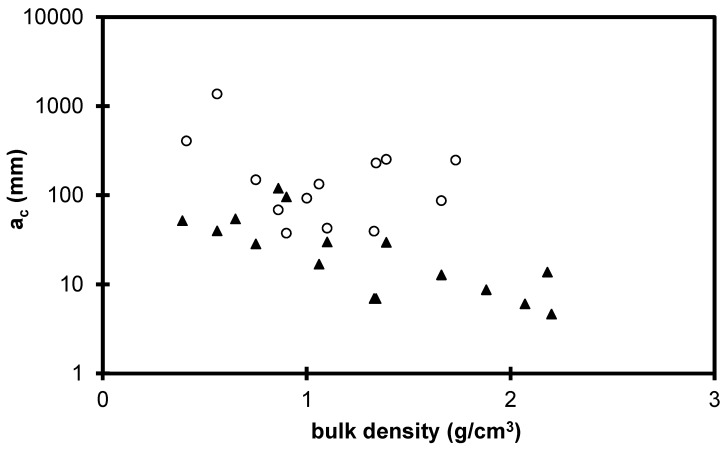
*a*_C_ evolution for the xerogels (o) and sintered aerogels (▲) *versus* the bulk density.

Figure 9 shows the evolution of the critical flaw size as a function of the bulk density for the xerogels and the sintered aerogels. For the set of sintered aerogels a_c_ is in the range 5–100 μm and decreases over the bulk density range.

In the literature, the macroporosity of sintered aerogel has been measured by porosimetry [28]. The results confirm that, during sintering, the macroporous volume is progressively reduced. We can thus associate the *a*_c_ decrease during the sintering to the larger pore size and macroporous volume decrease. The drying also strongly collapses the macroporosity [9], the mechanical results show also that the *a*_c_ decrease during the drying could be associated to the elimination of the larger pores.

For the different studied parameters (TMOS%, drying, sintering), *a*_C_ decreases when the macroporous volume decreases. However, the *a*_C_ values are much larger than the pore size; so the scale of critical crack extends to a large number of pores. The critical flaws, which lead to failure, are likely created during the mechanical tests. The failure occurs by progressively breaking bonds associating a large number of macropores. The macropores link into a macroscopic flaw, and catastrophic failure occurs when the size of the defects becomes critical.

### 2.2. Influence of Environment on Fracture Mechanisms

In silica glass, under a corrosive environment, the flaw size can increase with time and *v*, the crack velocity, is frequently expressed under the form of an empirical relation [22]:

(1) $$\text{v}=AK_{I}^{n}$$

where *A* and *n* are constants and *n* is called the chemical susceptibility factor. We could suppose a similar process in silica aerogels.

**Figure 10:**
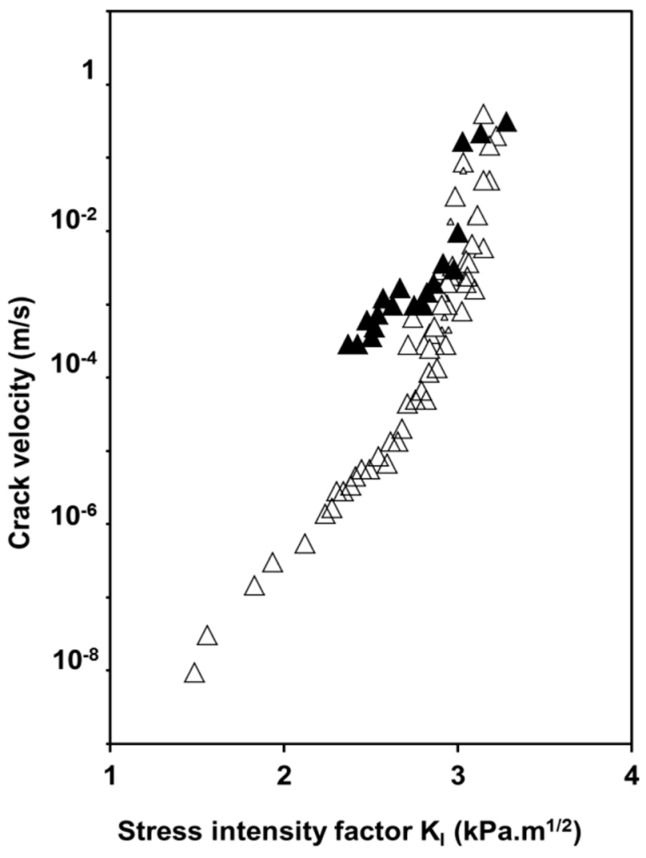
Evolution of the crack velocity as a function of the stress intensity factor for as prepared (Δ) and oxidized aerogel (▲) (ρ = 0.22 g·cm^−3^) under water vapour pressure of 1.4 KPa.

Crack growth studies investigated by DCDC (Double Cleavage Drilled Compression test, see “Experimental section”) revealed the same behavior for oxidized and as prepared aerogels under a humid atmosphere for a water vapor pressure about 1.4 KPa (50%Rh) (Figure 10) [32]. The chemical susceptibility factor (*n*) in the stress corrosion domain is identical for the two samples and equal to 15 indicating that the same chemical reaction is responsible for the crack propagation in both samples. The nature of the reaction is believed to be the same as for pure dense silica in alkaline solutions. Hydroxide ions cause a splitting of siloxane bonds.

**Figure 11:**
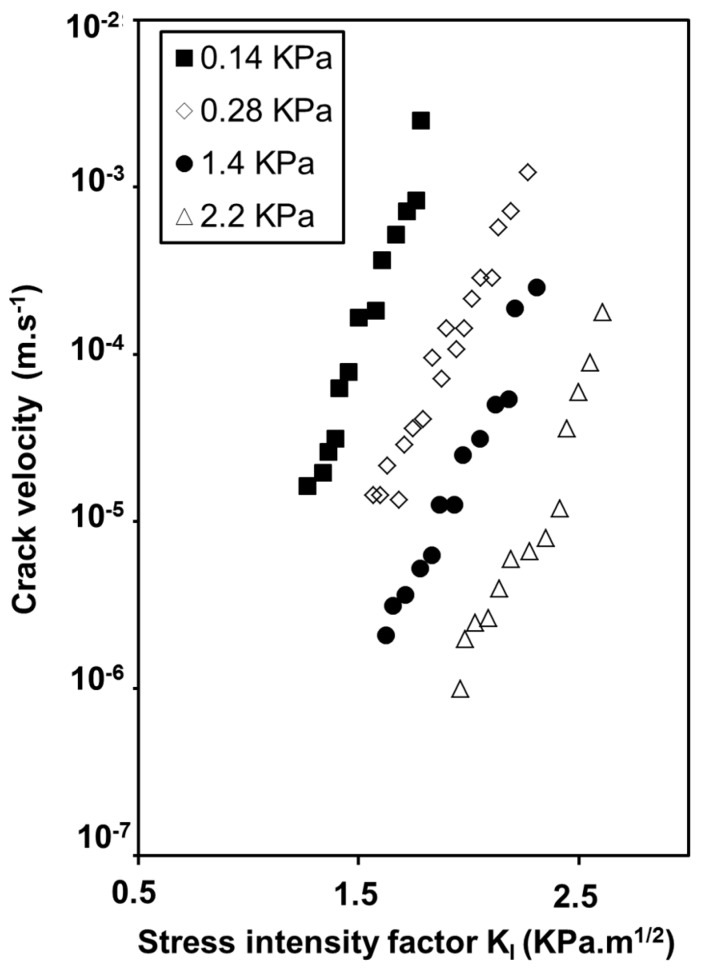
Evolution of the crack velocity as a function of the stress intensity factor under various water vapor pressure at ambient temperature—Oxidized aerogel (ρ = 0.22 g·cm^−3^).

Moreover, crack velocity is two orders of magnitude higher for the hydrophilic surface, which contains more silanol groups and, consequently, more adsorbed water and higher alkalinity. This result provides evidence of the influence of the OH content covering the aerogel surface on the dissociation of free water molecules and then on the formation of a critical flaw. Figure 11 illustrates the influence of the water vapor pressure on the crack velocity at 295 K [33]. Crack velocity curves shift to higher *K*_I_ values with the increase of water vapor pressure even when the slope of the curves remains the same.

**Figure 12:**
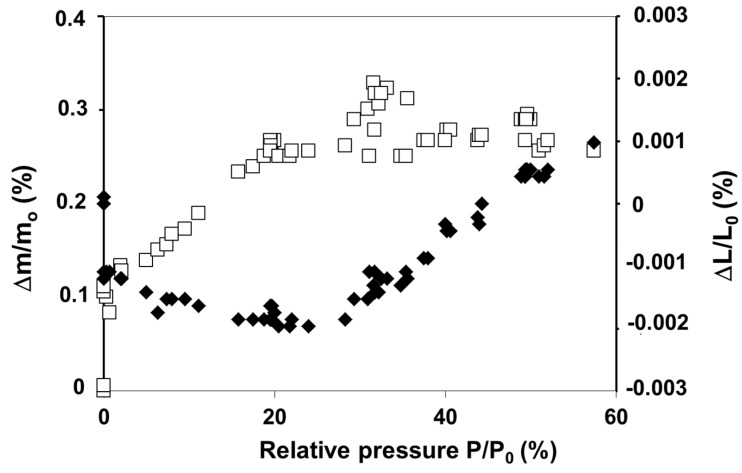
Dimensional (♦) and weight (☐) change measurements *versus* relative water vapour pressure for an oxidized aerogel (ρ = 0.22 g·cm^−3^).

This behavior is in the reverse order of that predicted by Wiederhorn’s model behavior which is described in literature in case of hygroscopic glass [34]. The fact is that aerogels adsorb water from the atmosphere [20,26]. A compressive stress originated from water adsorption contracts the backbone, the level of which depends on the relative water vapor pressure. In Figure 12, dimensional and weight change experiments performed under controlled water vapor pressure, show that sample expands or shrinks as a consequence of water adsorption or desorption inside the pores. Expansion or shrinkage levels depend on the OH content already adsorbed on the aerogel surface [35]. These sorption phenomena generate stresses which are high enough to modify the stress corrosion behavior. The swelling effect is likely due to a relaxation process, but we have no clear mechanism to propose.

**Figure 13:**
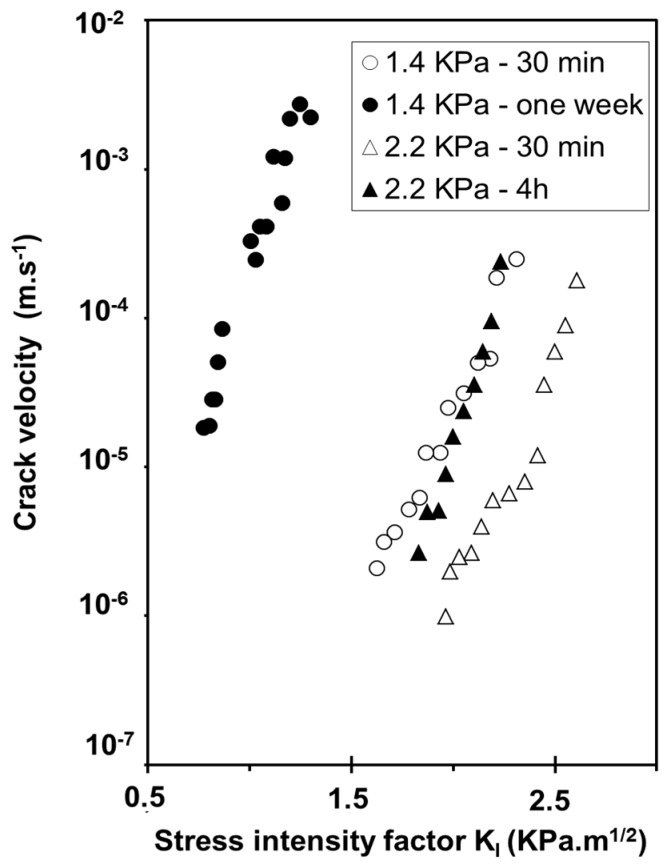
Evidence of the aerogel relaxation with increasing time when samples are in contact with water vapour at ambient temperature.

When time increases, curves shift to higher crack rates and lower *K*_I_ (Figure 13). The shift depends on observation time and on vapour vapor pressure. During adsorption of water, the aerogel firstly contracts when water begins to condense. The main effect is a compressive stress existing near the crack front which reduces the applied stress and then the crack velocity. When water condenses no more, the compressive stresses are relaxed. Siloxane bonds located at the crack tip are stressed and chemical reactions are enhanced inducing the curves shift toward higher rates.

**Figure 14:**
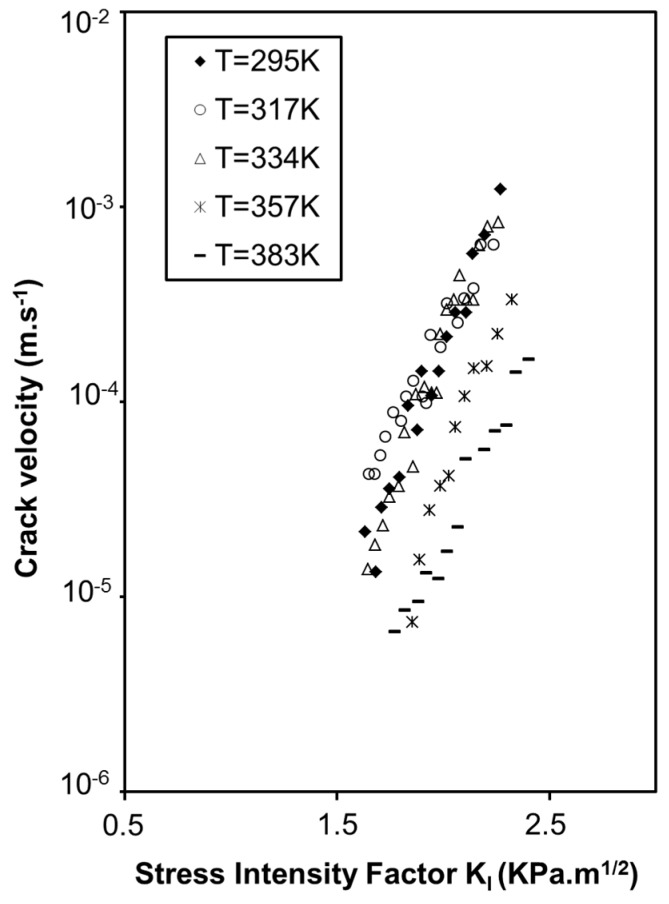
Evolution of the crack velocity as a function of the stress intensity factor at various temperature and for a constant water vapour pressure of 0.14 KPa—Oxidized aerogel (ρ = 0.22 g·cm^−3^).

The dependence in temperature of the sub-critical crack growth also shows an anomalous behavior (Figure 14). A decrease in crack velocity is observed when temperature increases [33].

In dense silica, this anomalous behavior has already been observed but is still not explained [36]. In silica aerogels, we propose that this result originates from capillary compression stresses, which are dependent of the temperature. All these results allow a better understanding of damages induced to aerogels during the supercritical drying process. Previous investigations have shown that, owing to the peculiar texture of the aerogel network, volume variation of the liquid due to thermal expansion but also to the supercritical fluid during depressurization could induce aerogel fracture. Previously calculated stresses induced by the supercritical drying procedure [37,38] were used to study a possible slow crack growth by a stress corrosion effect during the depressurization step. The chemical susceptibility factor calculated using the dynamic fatigue method shows that n is independent of vapor pressure [39] and is directly linked to the temperature by the relationship $n=\frac{E_{A}}{RT}$ [40]. *E*_A_ is the stress free activation energy of the SiO bond dissociation, *R* is the gas constant and *T* is the absolute temperature.

**Figure 15:**
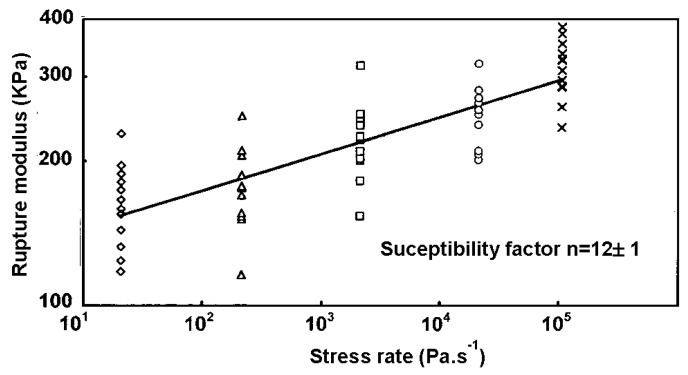
Plot of rupture modulus as a function of the stress rate of as-prepared aerogels.

The *n* value of 12 found for as prepared aerogels confirms that a stress corrosion effect is efficient under an alcoholic atmosphere (Figure 15) and thus during the supercritical drying.

**Figure 16:**
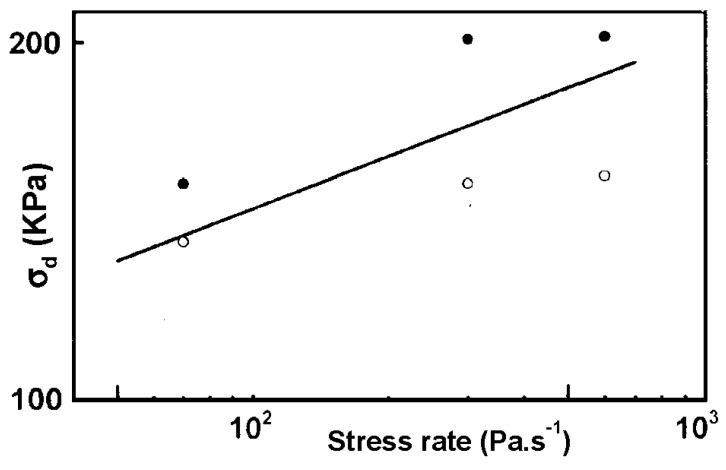
Plot of the stress σ_d_ as a function of the stress rate of as-prepared aerogels. Filled and open circles are respectively broken and save aerogels.

A value of about six should be found for the temperature 578 K. Using data reported in ref 44, with respect to the sample size, the authors estimated the maximum stress, σ_d_, suffered by the aerogel at the end of the depressurization. They reported the state (broken or save) of aerogels *versus t_D_*, the time passed to depressurize the autoclave from 13 MPa to 0.1 MPa. These results may be transformed to express the maximum stress as a function of the stress rate (Figure 16). The experiments may be regarded as equivalent to a dynamic fatigue test and as a guide to the eye, an arbitrary straight-line corresponding to *n* = 6 is drawn. Even so, it can be seen that there is an uncertainty in the proper value of the stress leading to aerogel fracture.

The results show that slow crack propagation by stress corrosion could be relevant during the supercritical drying procedure. As a consequence, a balance between a fast depressurization which implies large stresses and a slow depressurization rate which favors stress corrosion must be found to prepare monolithic aerogels.

## 3. Conclusions

Aerogel has specific properties because it is certainly the lightest solid material that has been produced. The possibility to fill and/or to sinter the porous structure is another way to enlarge the interest and applications of aerogels. However, because of this large pore volume, the mechanical properties of the dried gels are several orders of magnitude lower than those of the dense silica. The literature shows that the mechanical behavior is very close to that of porous glass. Aerogels are elastic and brittle materials, and porosity is the main parameter, which controls the mechanical features. In a more complete study, we show that two others parameters: pore size distribution and OH content are also significant to describe and understand the whole elastic and brittle behavior. For example, the defects (flaws) responsible for the fracture seem related to the macroporosity. The macropores link into a macroscopic flaw and catastrophic failure occurs when the size of the flaw becomes critical.

It has been shown [41] that the stress corrosion effect can lead to the failure of aerogels after several months under a low tension stress, and this effect is favored by the OH content of the gel. Stresses originated from the capillary condensation phenomenon inside the pores of the aerogel explain the anomalous temperature and water vapor pressure behaviors. In conclusion, porosity, pore size distribution and OH content are necessary parameters to have a complete description of the aerogels brittle behavior.

## 4. Experimental Section

### 4.1. Materials Synthesis

The silica gels selected in this study were made from tetramethoxysilane (TMOS) hydrolyzed under neutral (distilled water) or basic conditions (10^−2^ M, NH_4_OH). The gelling solution was poured into squared shaped “Pyrex” glass containers and aged two weeks at room temperature. The gels are transformed into aerogels by supercritical drying performed at 305 °C and 13 MPa [42]. Table 1 gives the bulk density of the aerogels sets *versus* the TMOS volume percent. Just after drying, aerogels are labelled “as prepared” and exhibit hydrophobic properties. A thermal treatment at 350 °C for 12 h allows removing their surface organic radicals. Oxidized aerogels are hydrophilic. Because aerogels rapidly adsorb water from the atmosphere, before each experiment, samples are outgassed for 24 h under primary vacuum and stored in evacuated desiccator. Before starting the experiment, they are maintained under controlled air moisture for 30 min.

The sintering of silica aerogels, which proceeds by viscous flow carried out at high temperature, (>1000 °C) has been described previously [26,27]. Depending on the duration of the heat treatment, the pores collapse and the bulk density increases up to the density of the silica glass 2.2 g·cm^−3^. These sintered aerogels samples covered porosity within the range 95%–0% (density between 0.1 and 2.2 g·cm^−3^).

Porosity can also be partially eliminated by a controlled and slow drying. These kinds of samples are labeled as xerogels [9]. These xerogel samples covered density between 0.5 and 1.6 g·cm^−3^.

Bulk density was determined measuring the samples weight and dimensions.

### 4.2. Mechanical Measurements

The elastic modulus (*E*) and the rupture modulus (σ) of the samples were measured by a three-point bending technique using an Instron 1196 mechanical testing machine with crosshead speed which could be varied from 10 µm/min to 100 mm/min [18,20,41]. The dimensions of the gels samples were 100 × 10 × 10 mm^3^.

Following the linear elastic fracture mechanics applied to gels [19,21], failure is assumed as the result of the stress concentration at the flaw tip. When stressed, a brittle material is characterized by a given value of the stress intensity factor *K_I_*. It is related to the applied stress σ_a_ and to the flaw dimension “a” by:

(2) $$K_{I}=\sigma_{a}Y\sqrt{a}$$

*Y* is a geometrical factor depending on the location and the flaw shape. The fracture occurs when *K_I_* reaches the specific value, *K*_IC_ (the toughness), which is an intrinsic property of the material. In this study, the toughness is measured by the Single Edge Notched Beam (SENB) technique [23,41]. The SENB method insure the introduction of sharp cracks with known dimensions. The saw cut notch was performed using a diamond saw of 50 µm width. The notch depth, “*a*”, has a length in such a way that the ratio *a*/*W* was between 0.25 and 0.3, where *W* is the height of the sample (≈10 mm). Crack propagation was studied with the Double Cleavage Drilled Compression test (DCDC) [32,43]. This technique needs specimen with dimensions 150 × 15 × 15 mm^3^ and a 4 mm diameter hole. The stress is gradually increased until the initiation from the hole of two symmetrical cleavage cracks. Experiments were carried out under controlled air moisture with a water vapor content range from 5% to 80%Rh in a glove box. The desired water vapor pressure was generated by bubbling dry nitrogen gas through water in a temperature-controlled water bath. Two heater elements were arranged around the sample for temperature control.

For brittle materials, the broad scattering of the mechanical strength values, is attributed to statistical nature of flaws. The sample strength distribution is usually analysed using Weibull’s statistical analysis [44,45]. For samples of identical dimensions, for which the effective volume is assumed constant, the failure probability is given by the relationship:

(3) $$P(\sigma )=1-\exp\;\left[-{\left(\frac{\sigma }{\sigma_{o}}\right)}^{m}\right]=P_{j}$$

The cumulative failure probability, *P_j_* has been calculated using the estimator $P_{j}=\frac{j-0.5}{N}$, where *j* is the order of the sample and *N* is the total number of samples. Weibull’s modulus, m characterizes the breadth of the strength distribution. σ_o_ characterizes the mean rupture modulus.

The literature shows that the strength modulus and fatigue lifetime of vitreous silica decrease in humid environments [46,47]. Thus, the mechanical fatigue because of the stress corrosion effect could limit their technological applications if the materials are under a permanent stress. Understanding the whole fracture behaviour of silica gels implies the study of the crack velocity as a function of the *K_I_* value.

**Figure 17:**
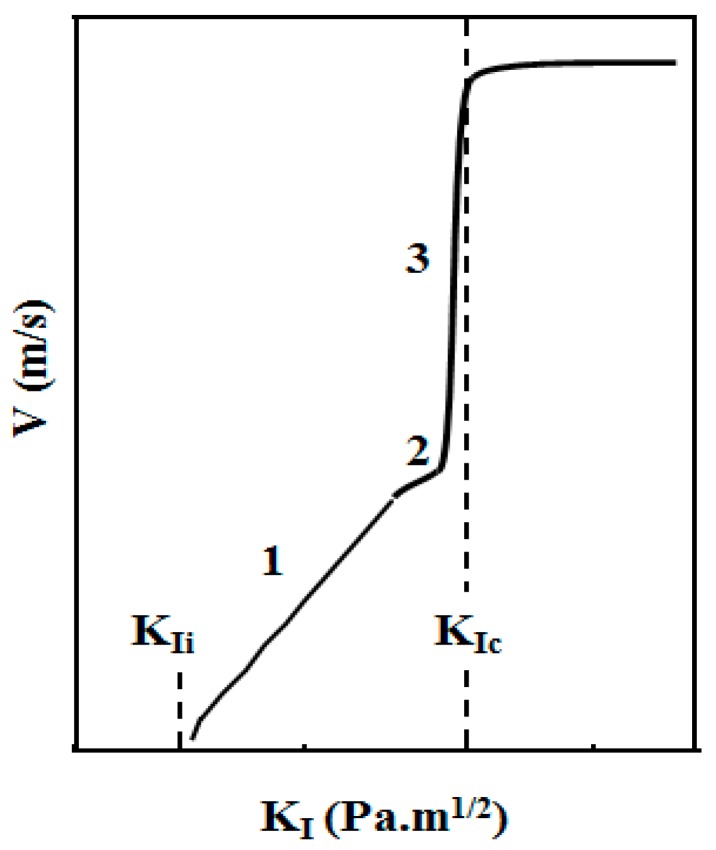
Schematic diagram of crack propagation speed *V* as a function of stress intensity factor *K_I_*. Region 1: the subcritical propagation; Region 2: the crack rate plateau; Region 3: high crack propagation.

In Figure 17, the crack velocity attains a constant value linked to the speed of the elastic waves [48], which are known to be slow in aerogels [49]. Regarding now the left side of the curve, it is clear that the crack velocity is low and the material may remain uncracked as long as *K_I_* is lower than *K_Ii_*. At *K_Ii_*, the crack velocity is equal to zero. The crack starts or stops when *K_I_* tends to *K_Ii_*. The life time of the material is mainly depending on the crack rate shown in the first part of the curve of Figure 17. However, the increase of *K_I_* may be obtained by raising the applied stress, the crack length or both parameters. In silica glass [22], the evolution of the crack velocity may be separated into three main domains (Figure 17).

The first domain is of interest since the crack evolves slowly. On the other hand, region 2 is often not observed. It corresponds to a diffusion limited reaction occurring at the crack tip. Since aerogels have porosity higher than 80% and are constituted by particles of about 50Å, the plateau region is not expected.

The crack propagates if the stress intensity factor becomes higher than *K_Ii_*. For *K_I_* > *K_I_*_i_ the crack propagates under the action of a stress corrosion effect due to chemical species reacting at the crack tip [47]. Bonds located at the crack tip are stressed and chemical reactions are enhanced. According to chemical kinetics, the activation energy *E_A_* is lowered when the curvature of the surface tip *r* or the stress σ increases [50]:

(4) $$E_{A}=E_{A}^{o}+\frac{B}{r}-C\sigma$$

where *B* and *C* are positive constants depending on the material and on the environment. Thus, the rate is increased and may be written by replacing σ with its *K_I_* expression:

(5) $$V\text{=}V_{\text{o}}\exp\left(-\frac{E_{A}^{o}+\frac{B}{\rho }-\frac{CK_{I}}{Y\sqrt{a}}}{RT}\right)$$

To analyse humidity or temperature effects on stress corrosion behaviours, a model based on the previous relationship was established by Wiederhorn [51].

(6) $$V_{I}=A{\left(\frac{p}{p_{o}}\right)}^{m}\exp\left(\frac{\left[K_{I}b-Q_{I}\right]}{RT}\right)$$

*V_I_* is the crack velocity in region *I*, *p* the water vapor pressure, *p_o_* the saturated water vapor pressure, *K_I_* the stress intensity factor and *T* the temperature. The parameters *A* and *m* are respectively a pre-exponential constant and the order of the reaction with water vapor as the reactant. *b* is a parameter which can be related to the activation volume and *Q_I_* is the activation energy of the chemical reaction.

However, it is noteworthy that the crack propagation in this range is more frequently expressed under the form of an empirical relation [22]:

(7) $$\text{v}=AK_{I}^{n}$$

where *A* and *n* are constants and *n* is called the chemical susceptibility factor.

If the chemical susceptibility factor can be obtained by dynamic fatigue experiments [52], the crack propagation measurements assume a test which allows expressing the crack velocity v as a function of the stress intensity factor *K_I_*. This test is called Double Cleavage Drilled Compression [43].

Moreover, when gels are submitted to a *K_I_* value in region 1, initial cracks propagate slowly as a function of time and can undergo a complete breaking of the material if the *K*_IC_ is reached. Knowing *n*, *m*, *K*_IC_, σ and the applied load σ_a_, it is possible to calculate a life prediction with a probability threshold [41].
